# Genetic Structure and Optimal Population Size of Wild and Mass-Selected Silver Pomfret (*Pampus argenteus*) in China: The Implications for Conservation and Selection Breeding Programs

**DOI:** 10.3390/biology14050534

**Published:** 2025-05-12

**Authors:** Mengya Xiao, Haipeng Yu, Yong Deng, Weixu Jiang, Yuanwen Zhang, Minglu Gao, Cheng Zhang, Jiabao Hu, Man Zhang, Shanliang Xu, Danli Wang, Yajun Wang

**Affiliations:** 1 National Engineering Research Laboratory of Marine Biotechnology and Engineering, Ningbo University, Ningbo 315211, Chinachengzhangcrab@163.com (C.Z.);; 2 College of Animal Science and Technology, Henan University of Science and Technology, Luoyang 471000, China; 3 Key Laboratory of Marine Biotechnology of Zhejiang Province, Ningbo University, Ningbo 315211, China; 4 Key Laboratory of Green Mariculture (Co-Construction by Ministry and Province), Ministry of Agriculture and Rural, Ningbo University, Ningbo 315211, China

**Keywords:** *Pampus argenteus*, population genetics, germplasm resources, microsatellite, mitochondrion

## Abstract

*Pampus argenteus*, a commercially significant marine species, faces genetic diversity challenges in selective breeding programs. This study implemented a comprehensive molecular strategy to evaluate genetic parameters in wild populations along China’s coast and three successive selected generations. The analysis utilized 19 highly polymorphic microsatellite loci, mt*COI*, and mt*D-loop* sequences to assess genetic structure. Compared with the wild populations, the average number of alleles (*N_a_*: 11.158–12.947), effective alleles (*N_e_*: 5.592–6.502), observed heterozygosity (*H_o_*: 0.626–0.665), expected heterozygosity (*H_e_*: 0.777–0.796), and allele richness (*A_r_*: 10.900–12.510) of the selected populations did not significantly decrease. In the mass selection of the three consecutive generations, the high genetic diversity of the selected population was successfully maintained. The effective population sizes of the first to third generations were estimated to be 83.7, 66.6, and 59.6, respectively. Population differentiation analysis showed minimal genetic divergence (FST = 0.0159–0.0326) with substantial gene flow, supported by clustering patterns indicating panmixia among wild populations from different geographical locations and among the selected populations. Notably, marginal decreases in diversity indices across generations suggest incipient genetic diversity decline, underscoring the imperative for systematic genetic monitoring. These findings validate the current breeding program’s efficacy in maintaining genetic variability while providing a framework for optimizing long-term selection strategies to prevent inbreeding depression.

## Simple Summary

This study systematically evaluated the genetic structure of three consecutive generations of selectively bred and wild populations of silver pomfret (*Pampus argenteus*) along China’s coast. The research aimed to address potential risks of genetic diversity loss during breeding programs while concurrently investigating the threat of inbreeding depression in these populations. Using 19 newly developed, highly polymorphic genetic markers and mitochondrial gene analysis, the selected populations showed no significant reduction in genetic diversity compared to wild populations, with effective population sizes maintained within 59.6–83.7. Strong gene flow (values > 1) and minimal genetic differentiation (0.0159–0.0326) between geographical groups confirmed successful integration of high-yield trait selection with genetic diversity preservation. However, a subtle decline in diversity was observed in selected populations, underscoring the necessity for ongoing monitoring to prevent inbreeding risks. The findings provide crucial insights for genetic improvement programs. They demonstrated that well-designed selective breeding can enhance aquaculture productivity and sustain population genetic health. This work establishes a scientific framework for balancing economic important characteristics with biodiversity conservation in marine fish breeding, offering practical strategies for sustainable aquaculture development.

## 1. Introduction

Selective breeding, as a core strategy with a long history, has always focused on optimizing specific traits of significant commercial value. Over decades of practice and exploration, this strategy has achieved remarkable results in the breeding fields of poultry and livestock, significantly enhancing their growth rate, production capacity, and reproductive performance [1,2]. In contrast, aquaculture species, with their high fecundity, short life cycles, and abundant morphological variation, have demonstrated a high degree of adaptability to intensive genetic improvement, thereby accelerating the pace of genetic progress [3,4]. However, it should be noted that intense selection pressure is often accompanied by a sharp reduction in the genetic variability of selected populations [5]. This phenomenon can be attributed to the selection of a few outstanding parents without pedigree information or to non-random mating between individuals with significantly different production performances [6,7].

Genetic diversity, the cornerstone of the long-term survival and evolutionary potential of species, is profoundly influenced by the interaction between selection and population history [8,9,10]. The reduction in diversity is closely linked to inbreeding depression, which not only weakens the adaptive capacity of species but also may constrain the potential for future improvement through artificial selection [11]. Significant losses in diversity have been observed in selected lines of various aquaculture species, including Pacific oysters (*Crassostrea gigas*) (Thunberg, 1793) [12], whiteleg shrimp (*Litopenaeus vannamei*) (Boone, 1931) [13], nile tilapia (*Oreochromis niloticus*) (Linnaeus, 1758) [14], rainbow trout (*Oncorhynchus mykiss*) (Walbaum, 1792) [5], European sea bass (*Dicentrarchus labrax*) (Linnaeus, 1758) [15], channel catfish (*Ictalurus punctatus*) (Rafinesque, 1818) [16]. Consequently, systematic evaluation of allelic diversity and population structure in artificially selected stocks becomes imperative to balance trait enhancement with conservation of evolutionary adaptability, thereby addressing dual challenges of inbreeding depression and non-directional allele frequency fluctuations [16].

Silver pomfret (*Pampus argenteus*) (Eupharasen, 1788) is an economically valuable marine fish [17,18,19]. With its high nutritional and medicinal value, it is deeply favored by consumers, and market demand continues to climb [20]. Effective genetic improvement of this species is of great significance for enhancing its commercial value. In fact, the economic value of *P. argenteus* is positively correlated with its size, especially during the Chinese New Year period, when the market price of large-sized *P. argenteus* can reach up to RMB 2000–3000 per kilogram. However, the market supply of large-sized *P. argenteus* remains tight. In this context, the selection breeding of *P. argenteus* with fast-growing traits undoubtedly holds broad commercial prospects. Previous studies have detailed the breeding program for fast-growing strain populations, and successfully conducted continuous group selective breeding of *P. argenteus* for three generations [21,22]. Currently, the parents of farmed *P. argenteus* in China mainly rely on populations from the East China Sea, and industrialized farming and genetic breeding have been achieved. However, populations from the Bohai Sea, Yellow Sea, and South China Sea have hardly been developed and utilized, and wild resources are facing challenges such as mixed germplasm resources, declined growth performance, and the gradual reduction in size and age of caught individuals.

In view of this, the present study aims to explore the differences in population genetic information between different generations of selected lines and between these lines and wild populations from different geographical locations. If the genetic differentiation within wild populations and within selected populations is relatively low, and the gene flow value between different populations exceeds 1, this suggests a significant level of gene exchange among wild populations from different geographical locations, as well as between different generations of selected populations. Here, in this study, we developed 19 highly polymorphic microsatellite markers and combined them with mt*COI* and mt*D-loop* markers to evaluate the genetic diversity of improved *P. argenteus* lines through mass selection for fast-growth traits, and a comparative analysis was conducted with wild populations from different geographical locations.

## 2. Materials and Methods

### 2.1. Sampling and DNA Extraction

**Figure 1:**
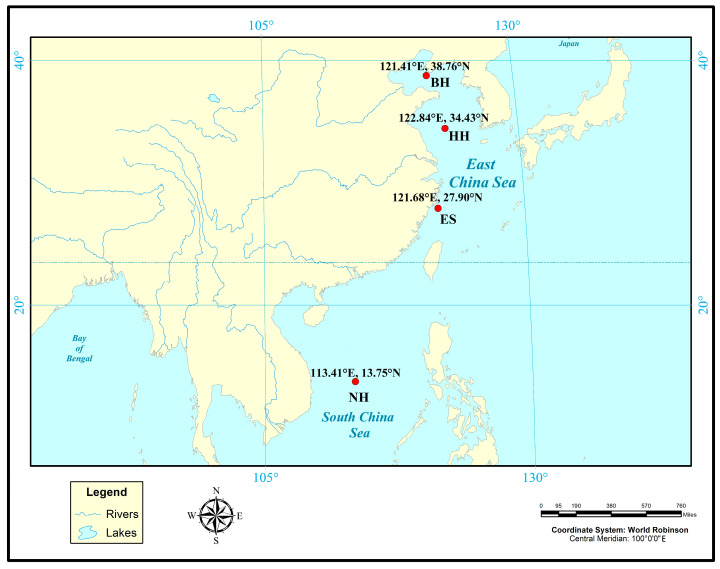
Distribution map of sampling points for wild *P. argenteus* populations.

In this study, the selected populations were obtained from different generations of strains selected, and the first, second, and third generations of the selected *P. argenteus* through continuous mass selection were named ES-G1, ES-G2, and ES-G3, respectively. Their daily management and feeding techniques were based on the previous research [22,23]. Hydrological parameters included daily water renewal rates of 100–220% with sustained microcurrent conditions, dissolved oxygen stabilized at 7 mg/L, and salinity maintained at 25 ppt. Thermal regulation-maintained water temperature between 15–28 °C, with parallel pH monitoring at 7.8–8.2. Nutritional provisioning followed a daily feeding regime equivalent to 3–4% of cohort biomass. Given the species’ documented photophobic behavior, diurnal photoperiod regulation was implemented using programmable LED arrays: 100 lx (06:30), 200 lx sustained from 10:30–16:30, stepped reduction to 50 lx (16:30–22:30), and nocturnal dimming to 5 lx post-22:30. Additionally, the wild populations were artificially captured by fishing vessels. The specific fishing populations were as follows: Bohai Sea (BH), Yellow Sea (HH), East China Sea (ES-G0), and South China Sea (NH) wild populations, and the coordinate information is shown in Figure 1. Forty individuals were randomly selected from each population. Muscle tissues were dissected from fresh specimens, preserved in 98% ethanol, and stored frozen at −20 °C until DNA extraction. The total genomic DNA from each sample was extracted from 30 to 50 mg of muscle tissue using the phenol-chloroform method [24]. The concentration of DNA was determined using a spectrophotometer. High-quality DNA was diluted to 50 ng/mL and stored at −20 °C.

### 2.2. Development of Microsatellite Markers and Screening

The HiSeq4000 high-throughput sequencing instrument (Illumina, San Diego, CA, USA) was used to perform “whole genome random sequencing” on the genomic DNA of *P. argenteus*. After filtering out adapters and low-quality sequences, the MISA v2.1 software was used to search for microsatellite loci. Then, primers were designed on the flanking sequence of the microsatellite using Primer Premier 5.0 (Premier Biosoft Interpairs, Palo Alto, CA, USA). We randomly designed 125 pairs of microsatellite primers based on primer design principles, and 19 pairs of highly polymorphic microsatellite primers were selected. The detailed information of the microsatellite primers is shown in Table S1, and they were uploaded to the GenBank database, with the accession numbers ranging from OQ376837 to OQ376855.

### 2.3. Design of mtCOI and mtD-Loop Primers

*P. argenteus* mitogenome (GenBank number: KJ754096.1) was used as a reference sequence for designing *COI* and *D-loop* primers. The sequence information of these two primer pairs is as follows:

*COI:*
F: 5′–GCATGAGCTGGTATAGTAGG–3′
R: 5′–GCTCAGACCATGCCCATATATC–3′
*D-loop:*
F: 5′–ACCATCCAGCTCATATCTTAATG–3′
R: 5′–GAATGATAGCTATGTCACGAG–3′

### 2.4. PCR Amplification

For the 19 highly polymorphic microsatellite loci, the total volume of the PCR reaction system was 25 µL, including 12.5 µL of 2 × Flash Hot Start MasterMix, 9.5 µL of ddH_2_O, 1.0 µL of nuclear genomic DNA, 1.0 µL of forward and reverse primers. The PCR amplification reaction program was as follows: 94 °C for 5 min of pre-denaturation; 94 °C denaturation for 45 s, annealing temperature for 30 s, 72 °C extension for 1 min, 35 cycles; finally, extend for 10 min at 72 °C. This study adjusted the annealing temperature of each PCR reaction program based on the parameters listed in Table S1. After that, the high-quality PCR products were used for SSR genotyping. Simultaneously, for the mt*COI* and mt*D-loop* primers, the total volume of the PCR reaction system was 25 µL, including 12.5 µL of Taq PCR MasterMix, 8 µL of ddH_2_O, 2 µL of nuclear genomic DNA, 1.25 µL of forward and reverse primers. The PCR reaction program was similar to that of microsatellites, except that the annealing temperature was 55 °C.

### 2.5. Genetic Diversity Analysis

Micro-Checker v.2.2.3 was used to detect silent alleles based on 1000 Monte Carlo simulations [25]. The population genetic parameters, including the number of alleles (*N_a_*), the number of effective alleles (*N_e_*), observed heterozygosity (*H_o_*), expected heterozygosity (*H_e_*), and Shannon information index (*I*), were calculated using POPGENE v.1.32 [26]. The private alleles function of the “poppr” package in R v4.1.3 software was used to calculate the number of private alleles (*N_p_*) [27]. The allele richness (*A_r_*) and inbreeding coefficient (*F_IS_*) were calculated using the R package “hierfstat (v0.5-11)” [28]. GENEPOP v.4.7.0 was used to detect the degree of deviation from the Hardy–Weinberg equilibrium (HWE) in each population [29]. The Markov chain parameters were set to 10,000 dememorizations, with a batch size of 20 and 5000 iterations per batch. Additionally, the effective population sizes were calculated using NeEstimator v.2.1 [30]. For mitochondrial molecular markers, the CLUSTAL W program of BioEdit v 7.0.9 was used to rearrange their sequences [31], and manual proofreading was performed. The data of the number of parsimony sites, the number of variable sites, the G/C content, and the genetic distance of these seven populations were analyzed using MEGA5 [32]. The genetic diversity indices of each population were calculated using DNAsp v5.10.01 and Arlequin v3.11, including polymorphic information content, nucleotide diversity, and haplotype diversity [33,34]. Median-joining (MJ) networks between these seven populations were constructed using Popart v1.7 [35].

### 2.6. Genetic Structure Analysis

The differentiation within and between populations was evaluated using molecular variance analysis (AMOVA) in Arlequin v.3.5 [36]. In addition, the pairwise *F_ST_* of the population was calculated using the diffCalc function in the R package “diversity”. To detect gene flow between populations, the parameter *N_m_* was calculated using paired *F_ST_* values. The genetic structure of seven populations was analyzed using the Bayesian clustering method as described in STRUCTURE v.2.3.4 [37]. The number of clusters (K) was set to 1 to 8, and each K value was repeated 10 times. The number of MCMC iterations and burn-in period were both set to 100,000 iterations. The delta K (∆K) value was estimated by STRUCTURE HARVESTER, and the maximum ∆K value corresponds to the optimal K value. Then, CLUMPP and DISTRUCT were used to estimate the average mixing coefficient for each K value, and the clustering results were visualized [38]. Principal Coordinate Analysis (PCoA) was performed using GenAlEx v.6.5. The R package “gplot” was used to construct a UPGMA phylogenetic tree based on Nei’s genetic distance. For mitochondrial molecular markers, AMOVA was used to evaluate genetic differentiation between populations. NTSYS-pc 2.1e was used to construct UPGMA phylogenetic trees based on genetic distance between populations and the shortest distance method [39].

## 3. Results

### 3.1. Genetic Diversity

In this study, 19 microsatellite loci were chosen to conduct genotyping from the seven wild populations. No significant scoring errors were observed at any of the loci, attributed to large allele dropout or stuttering issues. The *X*^2^ test for the Hardy–Weinberg equilibrium (adjusted by Bonferroni method) at 19 microsatellite loci indicated that 12 loci deviated from the Hardy–Weinberg equilibrium, indicating the presence of invalid alleles at these loci or genetic relationships between the selected individuals. Furthermore, there was not a significant linkage disequilibrium among the 19 microsatellite loci, indicating that allelic variations among these polymorphic microsatellites were independent of each other. *PIC* values of the 19 highly polymorphic loci ranged from 0.57 to 0.96 (*PIC* > 0.5), and the average *PIC* for all loci was 0.839 (Table S2). The genetic diversity of 19 microsatellite loci in seven populations of *P. argenteus* was shown in Table 1. Through biostatistical analysis, the results showed minimal differences in microsatellite diversity between wild populations and between selected populations; however, the *N_a_* and *N_e_* values of the four wild populations were significantly higher than those of the other three selected populations (*p* < 0.05). Simultaneously, the *H_O_* and *H_e_* values of the seven populations ranged from 0.611 to 0.674 and from 0.790 to 0.865, respectively, with no significant differences observed (*p* > 0.05). This indicated that the reduction in alleles did not immediately lead to a decrease in heterozygosity. Furthermore, all *H_O_* values were lower than those expected, and the *F_IS_* values varied from 0.165 to 0.266, indicating that heterozygous defects were common in both wild and selected populations. As for the *I*, the range of *I* for these seven populations was between 1.877 and 2.419, with *PIC* values ranging from 0.742 to 0.852. The *I* and *PIC* values of all populations were greater than 1.0 and 0.5, respectively, indicating a high degree of polymorphism (Table 1). The global HWE test showed that except for 1254F-3, 1254F-8, 1676F-5, 1676F-7, 1676F-1, 1676F-6, and 0460F-2, the remaining 12 loci deviated from the Hardy–Weinberg equilibrium to varying degrees. The HW test for each population found that a total of 104 loci were significantly deviated from the Hardy–Weinberg equilibrium (*p* < 0.05) in these seven populations. Among them, all loci in the NH population were significantly deviated from the Hardy–Weinberg equilibrium, and the HH, ES, and ES-G1 populations had the lowest number of significantly deviated loci, with 13 loci each (Table S3).

**Figure 2:**
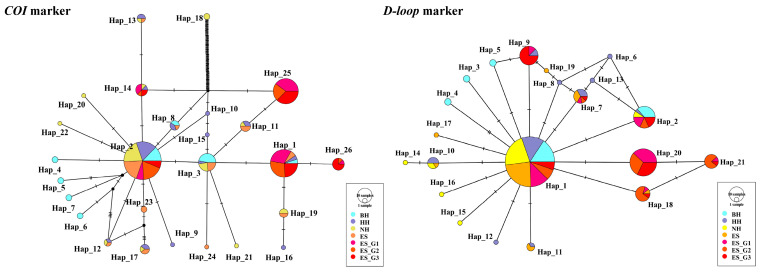
Median-joining (MJ) networks of wild and mass-selected populations of *P. argenteus* based on mitochondrial *COI* and *D-loop* markers.

Additionally, after sequence alignment, the mt*COI* gene fragment of 695 bp and the *D-loop* gene fragment of 418 bp were obtained from 239 *P. argenteus*, and their corresponding unique and shared haplotypes were identified as twelve and fourteen, nine and twelve, respectively. Meanwhile, the median-joining networks were constructed based on mt*COI* and mt*D-loop* molecular markers, dominated by a major haplotype shared by all seven populations (Figure 2). Among them, the analysis results based on the *COI* marker showed that the prevalence of haplotype 2 (Hap_2) among individuals was 33.47%, and the *D-loop* analysis results showed that the prevalence of haplotype 1 (Hap_1) among individuals was 48.12%. They were located in the center, while other low-frequency haplotypes were scattered around. Based on the molecular markers of mt*COI* and mt*D-loop*, *N_h_* of the wild populations was 21 and 14, respectively, while the unique haplotypes of the selected populations were two, and the rest were shared haplotypes. Detailed data can be found in Tables S4 and S5. On the whole, the average *H_d_* and *P_i_* of wild populations were higher than those of selected fast-growing strains. Similar to the results of microsatellite molecular marker analysis, the analysis based on these two markers showed that the seven populations were also at a high level of genetic diversity.

### 3.2. Effective Population Size

The effective population size of three consecutive generations of the fast-growing strain ranged from 59.6 to 83.7, and the *N_e-lin_* values of the selected populations were lower than those of the wild populations BH, NH, and ES, but similar to those of the HH population (64.1). More specifically, the *N_e-lin_* value of ES-G3 was relatively the lowest, at 59.6 (95% C.I. = 42.3–62.8), which was less than the actual number of parents (200 individuals). The *N_e-lin_* value of the wild population NH was relatively the highest, at 117.0 (95% C.I. = 86.6-Infinite), followed by the wild population ES at 105.0 (95% C.I. = 75.9-Infinite) (Table 2).

### 3.3. Population Structure Analysis

**Figure 3:**
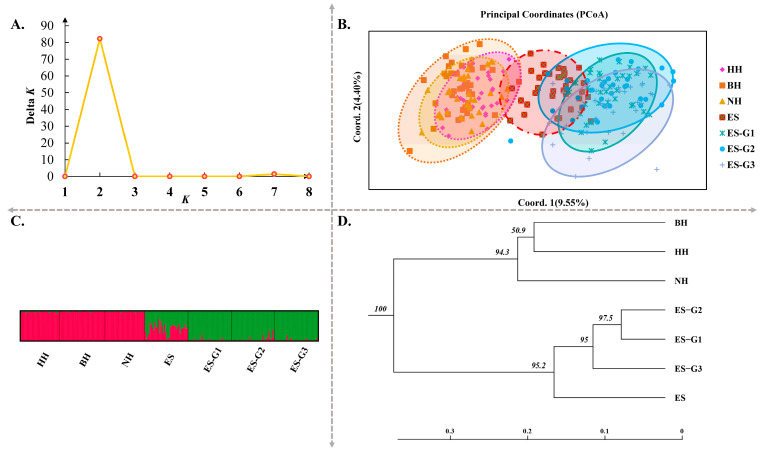
Analysis results of population genetics based on microsatellite molecular markers in wild and selected *P. argenteus* populations. Note: (**A**) evaluation values of delta *K*; (**B**) principal coordinates analysis (PCoA); (**C**) clustering analysis from STRUCTURE v2.3.4 software by 19 microsatellite loci; (**D**) construction of UPGMA phylogenetic trees based on Nei’s genetic distance.

The molecular analysis of variance (AMOVA) revealed these differences were observed between wild populations and between selected populations, with a significance level of *p* < 0.05 (Table 3). Additionally, the genetic differentiation coefficients between wild populations (excluding the ES population) and selected populations were significantly higher than those observed between selected populations (ranging from 0.0376 to 0.1052) and between wild populations. The trend of these results was basically consistent with the analysis results based on mt*COI* and mt*D-loop* molecular markers (Table S6). The PCoA based on the genetic distance matrix further revealed the genetic relationships between populations and individuals (Figure 3B). The HH, BH, and NH populations were found to be closely overlapped, and similarly, the ES-G1, ES-G2, and ES-G3 populations were also determined to be closely overlapped. Coordinate axis 1 (9.55%) and coordinate axis 2 (4.40%) were able to clearly distinguish these two overlapping regions. It was observed that the ES population overlapped within both of these major regions, which confirmed the high genetic similarity between the ES population and the overlapping regions (Figure 3B). During the cluster analysis of these seven populations, the studied samples were optimally divided into two theoretical groups due to the highly significant peak in the Δ K parameter curve at K = 2. The genetic composition of each cluster was clearly divided, and the degree of genetic mixing at the individual level was found to be very low (Figure 3A,C). It was established that there was a high level of mixing between wild individuals and between selected individuals, leading to their classification into two clusters.

**Figure 4:**
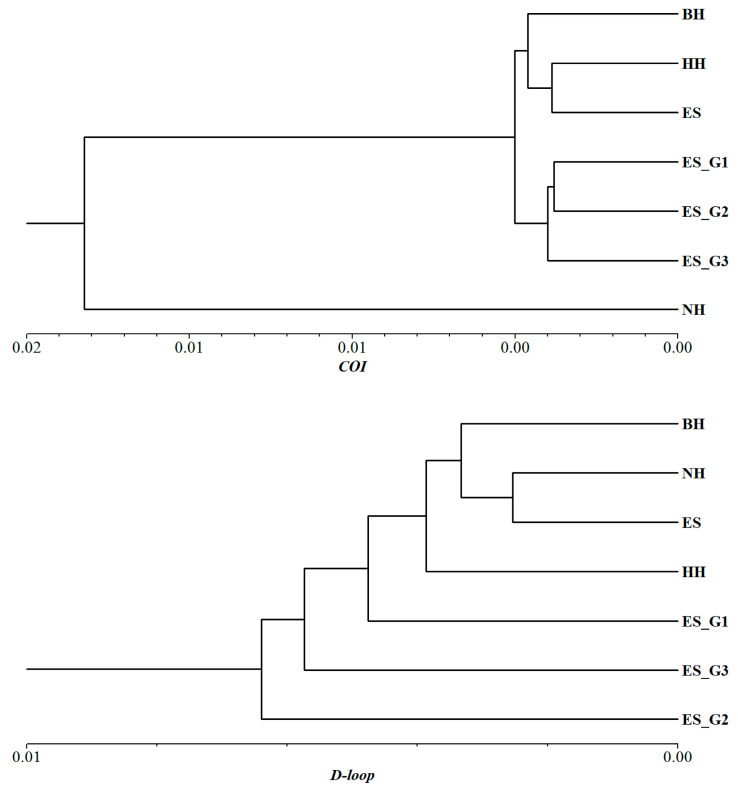
UPGMA phylogenetic trees constructed based on genetic distance of seven *P. argenteus* populations using mitochondrial *COI* and *D-loop* molecular markers.

In order to further understand the relationships among different populations, UPGMA phylogenetic trees were constructed based on Nei’s unbiased genetic distance (Figure 3D and Figure 4). The topological structure of the tree based on microsatellite markers revealed that the seven populations were clustered into two major branches. The wild populations BH, HH, and NH were placed on one separate branch of the clustering tree, while the three consecutive generations of selected populations, along with the ES population, were clustered on another branch. Furthermore, the selected populations were further subdivided into three branches based on the selected strain. At the same time, it was observed that the differentiation between adjacent generations in three consecutive generations of selected populations became increasingly evident. Similar conclusions were also confirmed by the results of PCoA. The topological structure of the UPGMA tree based on mt*COI* was similar to that of microsatellite markers. However, the topological structure based on mt*D-loop* molecular marker found that the wild population was still clustered first, and then the selected population gradually gathered with them into a large group.

## 4. Discussion

### 4.1. Genetic Diversity Among Populations

In aquaculture, the question of whether the genetic diversity of selected strains would be lost after long-term or intensive selection breeding has attracted much attention. This study revealed that after three generations of mass selection, the genetic diversity of the fast-growing strain did not show significant loss. The haplotype diversity of the selected population was only slightly lower than that of the wild population, indicating a slight downward trend in genetic diversity under artificial selection. This result strongly demonstrated the effectiveness of current breeding strategies in maintaining genetic variability. The number of haplotypes observed in the wild populations were relatively small compared to the selected populations. Based on this inference, the decrease in the number of haplotypes should not be simply attributed to inbreeding, but is more likely to be a reflection of the natural distribution of haplotype diversity in *P. argenteus*. These conclusions were similar to the results of other studies [6,40]. However, it is worth noting that the number and diversity of haplotypes in the selected population have decreased, which were consistent with the slight decreasing trend of *N_a_*, *I*, *H_o_*, *H_e_*, *A_r_*, and *PIC*, suggesting that mass selection may have a potential impact on the genetic variation of fast-growing strains. Similar conclusions were also reached in studies of other species [6,40,41,42]. At the population level, the loss of variation in microsatellite alleles may be seen as a factor in reducing potential key functional genetic variations in the genome, and may have a negative impact on the adaptation of offspring due to the disappearance of rare alleles [43]. Additionally, the potential loss of genetic diversity during the breeding process has been proven to be an unavoidable phenomenon in both theory and practice [44]. In this study, *N_a_* content did not show a significant downward trend among three consecutive generations, indicating effective management in previous selection breeding and also meaning that there was no need for excessive intervention to avoid unintentional loss of *N_a_*. In addition, the average heterozygosity level of the wild population was observed to be higher compared to three consecutive generations of selected populations, and the expected heterozygosity of all populations was found to exceed the actual observed heterozygosity. It was found that rabbitfish (*Siganus oramin*) [45], rock bream (*Oplegnathus fasciatus*) [46], and red Swamp Crayfish (*Procambarus clarkii*) [47] showed a similar pattern, revealing a certain degree of inbreeding among wild populations. Here, we speculated that the wild populations may have broken through the geographical limitations between different sea areas due to their excellent swimming ability, thereby increasing the influence of inbreeding to a certain extent.

Additionally, it was found that both wild populations and three consecutive generations of *P. argenteus* selected populations showed generally higher genetic variation within populations than between populations. These seven populations exhibited high intra-population variability and low inter-population variability. The high genetic variation within the population reveals abundant genetic diversity within the population [44]. According to research results, the genetic diversity of each wild population was higher than that of the selected populations. This high level of diversity further confirmed the effectiveness of selective breeding programs and appropriate genetic management of fast-growing strains. Meanwhile, this also meant that the number of effective individuals contributing to the population did not decrease (Table 2). Overall, the fast-growing strains of *P. argenteus* did not lose alleles due to random genetic drift, and the selection pressure of excellent growth traits had no effect on the gene pool (Neigel, 1997 [48]). Therefore, these findings provided valuable information for the selective breeding program of fast-growing strains being implemented, as selecting individuals from highly diverse populations was crucial for building a broad base population with excellent genetic diversity traits.

### 4.2. Effective Population Size

The fluctuations in *N_e-lin_* were often influenced by agricultural limitations, which in turn limited the contribution of parents to offspring [49]. In the aquaculture practices, the reduction in *N_e-lin_* was often caused by factors such as insufficient parental quantity, imbalanced gender ratios, uneven contributions of gametes, and differences in gamete survival [50]. Meanwhile, the strategy of selecting parents based on commercial traits may have further exacerbated the reduction in *N_e-lin_* in large-scale aquaculture populations, as in this case, excellent parents often come from a few families [11]. This study observed that, as the number of generations of genetic breeding increased, the *N_e-lin_* values of ES-G1, ES-G2, and ES-G3 populations showed a gradually decreasing trend. This phenomenon may be closely related to the uneven contribution of parents between different generations, the scarcity of parents, and the increasing selection pressure [22]. It is worth noting that *P. argenteus* is a fish that lays eggs twice a year, with a breeding cycle of up to two months [51]. During this period, asynchronous gonadal development between parent fish occurs from time to time [52], which undoubtedly increases the difficulty of reproductive management. In addition, due to the difficulty in distinguishing male and female individuals in the phenotype of *P. argenteus*, and the lack of precise molecular markers for gender identification, it is difficult to achieve optimal matching of male and female ratios in practical operations [22,23]. These factors combined may have further widened the differences in reproductive success rates. Given this, maintaining a sufficiently large and effective population size is of crucial importance in mitigating the negative effects of inbreeding and preventing the loss of genetic variation. Of course, to achieve this goal, in addition to increasing the use of parent fish, improving the reproductive performance of the population is also a promising option [21,22].

### 4.3. Genetic Structure Among Populations

Advantage of diversity largely depends on the degree of differentiation between populations [53]. *F_ST_* is one of the most widely used indicators for evaluating the degree of heterogeneity between populations [54]. In this study, there were moderate to high levels of heterogeneity between wild and selected populations. This may be due to the lack of significant genetic structure or population subdivision within wild populations and within selected populations, leading to frequent gene exchange among individuals within these respective populations. The gene flow parameters analyzed in this study also support this conclusion. Although it was found through pairwise analysis of *F_ST_* and genetic distance that the heterogeneity from the first generation to the third generation gradually increased, the relatively small difference indicated that the genetic variation of the selected population may tend to stabilize under selection pressure [55]. Additionally, there was not much difference in heterogeneity between different geographical locations within wild populations, indicating that gene exchange between wild populations was not affected by factors such as geographical location and natural selection pressure. Their high genetic similarity may be attributed to their strong swimming ability and common origin.

Through cluster analysis of genetic distance, we observed that wild populations in the Yellow Sea, Bohai Sea, and South China Sea clustered into one branch, while wild and farmed populations in the East China Sea formed another independent branch. This discovery further confirmed that these populations may have shared highly similar genetic information during their evolutionary process. It is worth noting that the interconnection between the two major branches not only revealed the dominant position of the wild populations in the development of selected populations in the East China Sea, providing the main source of alleles, but also emphasized the close genetic relationships between different wild populations. This conclusion was consistent with our previous research findings of high gene flow and low genetic differentiation. This phenomenon implied that gene introgression between wild and selected populations was extremely common, which posed significant management challenges for the protection of genetic resources in the wild and selected populations.

## 5. Conclusions

This study first targeted the development of 19 highly polymorphic microsatellite loci, and based on these microsatellite and mitochondrial molecular markers, conducted in-depth exploration of the genetic diversity and population structure of wild and mass-selected populations. The research results showed that the high haplotype diversity and low nucleotide diversity were exhibited among different geographical populations. As the breeding process continues to deepen, the genetic diversity of the selected population gradually decreases. In addition, the genetic differentiation levels between wild populations in different geographical locations were low, and the gene flows were high, indicating that mating between wild populations may not be affected by factors such as geographical distance, watershed connectivity, or environmental differences. The genetic differentiation and gene flow trends of the selected populations were similar to those of the wild populations, reflecting the effectiveness of management measures in the mass selection process. Overall, this study conducted a comprehensive and detailed evaluation of the population genetics of *P. argenteus* in the four major coastal areas of China. Both the wild and the mass-selected populations maintain a high level of genetic diversity, indicating that wild germplasm resources can be further developed and utilized reasonably. At the same time, it also indicates that the current *P. argenteus* fast-growing strain can be mass-selected according to the breeding program.

## Figures and Tables

**Table 1:** Genetic diversity of the wild and selected *P. argenteus* populations.

Populations	*N_a_*	*N_e_*	*I*	*H_o_*	*H_e_*	*N_p_*	*A_r_*	*F_IS_*	*PIC*
HH	16.895	10.847	2.419	0.663	0.882	35	17.050	0.242	0.852
BH	13.000	8.634	2.192	0.611	0.844	23	13.260	0.266	0.817
NH	15.316	9.041	2.330	0.652	0.865	39	15.340	0.247	0.838
ES	16.790	9.323	2.315	0.674	0.854	53	16.650	0.213	0.826
ES-G1	11.158	5.919	1.877	0.665	0.777	12	10.900	0.165	0.742
ES-G2	12.947	6.506	1.994	0.626	0.796	24	12.510	0.224	0.766
ES-G3	11.947	5.592	1.899	0.639	0.790	34	11.500	0.199	0.753

Note: *N_a_*: number of alleles; *N_e_*: effective number of alleles; *I*: Shannon information index; *H_o_*: observe heterozygosity; *H_e_*: expected heterozygosity; *N_p_*: number of private alleles; *A_r_*: allele abundance; *F_IS_*: proximity coefficient; *PIC*: polymorphic information content.

**Table 2:** List of effective population size for each population.

Populations	The Number of Samples	The Effective Population Sizes (*N_e-lin_*)	95% C.I. (Lower–Upper)
HH	32	64.1	49.0–859.2
BH	38	90.5	79.8–353.6
NH	33	117	86.6–Infinite
ES	36	105	75.9–Infinite
ES-G1	36	83.7	66.7–234.3
ES-G2	36	66.6	41.5–110.6
ES-G3	36	59.6	42.3–62.8

**Table 3:** Analysis of molecular variances (AMOVA) of microsatellites and mitochondrial molecular markers for the wild and selected *P. argenteus* populations.

Markers	Source of Variation	*d.f.*	Sum of Squares	Variance Components	Percentage of Variation	F-Statistics
Microsatellite loci	**Among wild population**					
	Among populations	3	110.788	36.92936	4.45	0.04453 **
	Among individuals/within population	135	1385.381	10.26208	23.36	
	Within individuals	139	866.000	6.23022	72.19	
	Total	277	2362.169	53.42165		
	**Among selected population**					
	Among populations	2	45.056	22.52778	2.45	0.02447 **
	Among individuals/within population	105	937.931	8.93267	18.19	
	Within individuals	108	661.500	6.12500	79.36	
	Total	215	1644.486	37.58545		
mt*COI*	**Among wild population**					
	Among populations	3	25.001	0.14773 Va	3.93	0.03929 **
	Within populations	124	447.881	3.61195 Vb	96.07	
	Total	127	472.883	3.75967		
	**Among selected population**					
	Among populations	2	4.241	0.02559 Va	2.09	0.02089 *
	Within populations	105	125.917	1.19921 Vb	97.91	
	Total	107	130.157	1.22479		
mt*D-loop*	**Among wild population**					
	Among populations	3	3.044	0.01764 Va	3.87	0.03872 *
	Within populations	127	55.612	0.43789 Vb	96.13	
	Total	130	8.656	0.45553		
	**Among selected population**					
	Among populations	2	9.315	0.10228 Va	9.49	0.09491 **
	Within populations	105	102.417	0.97540 Vb	90.51	
	Total	107	111.731	1.07767		

Note: * represents *p* < 0.05; ** represents *p* < 0.01.

## Data Availability

The authors declare that the original data of this study are available from the corresponding authors.

## Supplementary Materials

The following supporting information can be downloaded at https://www.mdpi.com/article/10.3390/biology14050534/s1, Table S1. Nineteen highly polymorphic microsatellite primers developed for *P. argenteus*; Table S2. The results of Hardy–Weinberg test for the 19 highly polymorphic microsatellite loci; Table S3. The results of Hardy–Weinberg test for the wild and selected *P. argenteus* populations; Table S4. Genetic diversity and haplotype frequency of mt*COI* sequences observed among the wild and selected *P. argenteus* populations; Table S5. Genetic diversity and haplotype frequency of mt*D-loop* sequences observed among the wild and selected *P. argenteus* populations; Table S6. Paired *N_m_* and *F_st_* values of the wild and selected *P. argenteus* populations.
